# Valorization of Olive Stones: Chemical Composition and Bioactivity

**DOI:** 10.3390/ph19030447

**Published:** 2026-03-10

**Authors:** Bruna Nunes, Naiara Fernández, Andreia Bento Silva, Ana Partidário, Joana Marto, Elisabete M. C. Alexandre, Sofia Lourenço, Ana Teresa Serra, Maria Rosário Bronze, Noélia Duarte, Ana Margarida Rodrigues

**Affiliations:** 1 Research Institute for Medicines (iMed.ULisboa), Faculdade de Farmácia, Universidade de Lisboa, Avenida Professor Gama Pinto, 1649-003 Lisboa, Portugal; brunanunesbss@outlook.com (B.N.); abentosilva@ff.ulisboa.pt (A.B.S.); jmmarto@ff.ulisboa.pt (J.M.); tserra@ibet.pt (A.T.S.); mbronze@ibet.pt (M.R.B.); 2 iBET, Instituto de Biologia Experimental e Tecnológica, Av. da República, Quinta do Marquês, 2780-157 Oeiras, Portugal; naiara.fernandez@ibet.pt; 3 Instituto Nacional de Investigação Agrária e Veterinária (INIAV), I. P., Av. da República, Quinta do Marquês, 2780-157 Oeiras, Portugal; ana.partidario@iniav.pt; 4 Perfinox, Zona Industrial Lameiradas Lt 1 Mansores, 4540-423 Arouca, Portugal; elisabete.alexandre@perfinox.pt; 5 Sovena Portugal—Consumer Goods, S.A., Rua Doutor António Loureiro Borges 2, Edifício Arquiparque 2 Miraflores, 1495-131 Algés, Portugal; sofia.lourenco@sovena.pt; 6 Instituto de Tecnologia Química e Biológica Antonio Xavier, Universidade Nova de Lisboa, Av. da República, 2780-157 Oeiras, Portugal

**Keywords:** olive stone, microwave-assisted extraction, phenolic compounds, fatty acids, antioxidant, antiproliferative

## Abstract

**Background/Objectives**: The consumption of olive oil and olives has been steadily increasing, leading to growing interest in the sustainable management of by-products such as olive stones. This work aimed to contribute to the development of valorization strategies by studying the chemical composition and biological potential of olive stone extracts, using both conventional and eco-friendly extraction methods with various solvents. **Methods**: Several extracts were prepared and chemically characterized regarding their fatty acid and phenolic profiles by GC-FID and HPLC-DAD-MS/MS, respectively. Their antioxidant, cytotoxic and antiproliferative activities were also evaluated. **Results**: n-Hexane Soxhlet extract yielded higher concentrations and a broader range of fatty acids when compared to the chloroform-methanol Folch extract. Oleic, palmitic, and linoleic acids were the predominant fatty acids in the extracts. A large variety of phenolic compounds were identified in extracts obtained by microwave-assisted extraction (MAE), including several flavonoids, small phenolic compounds, secoiridoids (nuezhenide and oleuropein), and hydroxytyrosol. MAE hydroalcoholic extracts showed high total phenolic content (TPC), antioxidant activity by the oxygen radical absorption capacity (ORAC) and hydroxyl radical scavenging capacity (HOSC) assays. Moreover, the aqueous ethanol (50%) and aqueous methanol (80%) extracts displayed low cytotoxicity toward the non-malignant Caco-2 cell line (IC_50_ values of 1.29 and 1.40 mg/mL, respectively), while both induced complete loss of viability in the HT-29 human colon adenocarcinoma cell line at 0.63 mg/mL. **Conclusions**: These findings highlight the potential of olive stone as a valuable source of bioactive compounds with antioxidant and selective antiproliferative properties. The results support their further exploration in the development of sustainable valorization strategies for olive industry by-products.

## 1. Introduction

The production of olive oil and table olives are relevant industrial and socioeconomic activities, particularly in the Mediterranean countries. In recent decades, the global consumption of olive oil has experienced a consistent upward trend, largely attributed to its organoleptic characteristics and increasing consumer awareness of health-promoting properties [1]. Numerous studies have highlighted the beneficial effects of olive oil consumption, particularly its cardioprotective role and its potential in reducing the risk of various chronic diseases [2,3,4,5]. According to the International Olive Oil Council, olive oil production has tripled over the last 60 years. Provisional data from 24/25 points to a production of approximately 3.57 million tonnes and an estimated production of around 3.44 million tonnes for the 25/26 crop year [6,7]. This production has been paralleled by a significant increase in olive-related by-products, some of which have considerable environmental impacts. Olive pomace, i.e., the solid residue after oil extraction, olive leaves, which are often discarded during harvesting and processing, and olive stones are generated in large quantities across the olive oil and table olive industries. While olive leaves and pomace have been widely studied regarding their phytochemical profile and bioactivities [8,9,10,11,12,13], olive stones remain comparatively underexplored [13,14,15,16,17].

In the olive oil industry, olive stones can be recovered from solid waste by filtration. From the pitted table olive industry, they are recovered by separation of the pulp [14]. Despite traditional olive stones being regarded as a low-value by-product, recent advances in green chemistry and bioeconomy principles sparked interest in investigating olive stones as a source of high-value compounds with potential applications in nutraceuticals, pharmaceuticals, and cosmetics [14]. Olive stones represent about 18–25% of the total olive weight and are mainly composed of lignocellulosic material (i.e., cellulose, hemicellulose, and lignin), containing also lipophilic compounds, such as fatty acids and sterols, and phenolic compounds (e.g., flavonoids, phenolic acids and alcohols, and secoiridoids) [18,19,20,21,22,23,24,25]. These compounds have been associated with various biological activities, including antioxidant, antimicrobial, anti-inflammatory, and antiproliferative [26].

The efficient extraction of phenolic compounds is also a key factor for the development of sustainable valorisation strategies. The use of green extraction methods—including microwave-assisted extraction, ultrasound-assisted extraction, pressurized liquid extraction, enzyme-assisted extraction and supercritical fluid extraction—has shown increasing popularity as an alternative to conventional techniques (e.g., solid-liquid extraction, Soxhlet extraction, liquid–liquid extraction, percolation and maceration), enhancing extraction efficiency, while being less time-consuming and environment-friendly as they do not require large volumes of solvents [24]. In microwave-assisted extraction (MAE), the mixture is heated by microwave radiation, facilitating the dissolution of the compounds into the chosen solvent. It is characterized by the use of small solvent amounts and its less time-consuming [27,28]. This method has been reported to enhance the extraction yield of phenolic compounds in plant matrices compared to conventional methods [29,30]. Furthermore, according to a life-cycle assessment, the use of MAE in pectin extraction from orange peel was reported to reduce the environmental impact by approximately 75% compared with the conventionally used acid-assisted thermal process [31]. All these characteristics highlight this method’s potential for the development of an environmentally friendly and cost-effective valorisation strategy of olive stones.

The present study aims to contribute to the sustainable valorisation of olive industry by-products by characterizing the chemical composition and biological activity of olive stone extracts. The main goals were to compare the efficacy of different extraction techniques (both conventional and MAE as a green alternative) by analyzing the extracts’ chemical profiles using gas chromatography coupled to a flame ionization detector (GC-FID) and high-performance chromatography coupled to diode array and mass detectors (HPLC-DAD and HPLC-DAD-MS/MS). Cytotoxicity of the extracts was evaluated in the Caco-2 cell model. The antioxidant activity was assessed using Oxygen Radical Absorbance Capacity (ORAC) and Hydroxyl Radical Scavenging Capacity HOSC assays, and antiproliferative activity was evaluated using the HT-29 human colorectal adenocarcinoma cell line. These evaluations are particularly relevant in the context of colorectal cancer, where oxidative stress plays a key role in promoting DNA damage, chronic inflammation, and tumour initiation/progression. Excess reactive oxygen species (ROS) drive genomic instability and support cancer development, while antioxidants may help mitigate oxidative damage and inflammation, thereby offering protective or therapeutic potential against colorectal cancer [32]. Thus, assessing the antioxidant capacity and antiproliferative effects of these extracts provides valuable insights into their possible role in countering oxidative stress-related mechanisms in colorectal carcinogenesis.

## 2. Results and Discussion

### 2.1. Lipid Fraction: Extraction and Characterization

To better characterize olive stones, the lipid fraction and polar extracts of this by-product were prepared. To obtain the lipid fraction, the Soxhlet and Folch methods were employed, using *n*-hexane and a chloroform–methanol (2:1) mixture as the extraction solvents, respectively. Although similar extraction yields were achieved (0.93 ± 0.09 and 0.98 ± 0.06% dry weight basis (DWB), respectively, slight variations were observed regarding the fatty acid profile. The Soxhlet extract contained a broader range of fatty acids, and four unsaturated long-chain fatty acids, namely, palmitoleic acid (*cis*-7), eicosenoic acid (*cis*-5), eicosadienoic acid and docosadienoic acids were found exclusively in this extract (Supplementary Tables S1 and S2). In contrast, vaccenic acid, also a long-chain and unsaturated fatty acid, was detected only in the Folch extract. Even though there were slight differences in the profiles obtained from both extraction methods, oleic acid was the major fatty acid identified, followed by palmitic acid and linoleic acid, consistent with previous studies [22,33,34]. These findings also align with the fact that these fatty acids are also the most predominant in olive oil, particularly oleic acid [35,36]. Furthermore, both extracts showed a higher concentration of unsaturated acids, with the Soxhlet extract containing higher amounts of both monounsaturated FA (363.03 μg/mg) and polyunsaturated fatty acids (65.39 μg/mg), compared to the Folch extract (218.65 and 29.54 μg/mg, respectively) (Supplementary Tables S1 and S2). Saturated fatty acids were present in relatively low concentrations in both extracts (110.74 and 77.72 μg/mg in the Soxhlet and Folch extracts, respectively). The fatty acid profile of olive stones is similar to that of olive oil, dominated by monounsaturated FA oleic acid (55–83%). Other fatty acids commonly present in olive oil include linoleic acid (3.5 to 21%), palmitic acid (7.5 to 20%). The higher proportion of unsaturated fatty acids compared to saturated fatty acids is also in accordance with the literature [22,33,34]. The lower concentration of saturated fatty acids is a good indicator of a promising lipid profile for the application and valorization of this by-product due to its positive impact on cardiovascular health [37].

### 2.2. Polar Compounds Extraction and Total Phenolic Content (TPC) Determination

**Figure 1:**
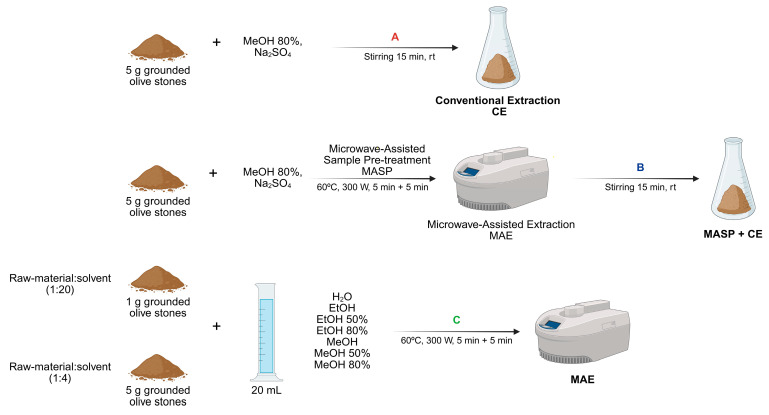
Methods employed for the extraction of polar compounds from olive stones: (A) Conventional extraction (CE); (B) microwave-assisted sample pre-treatment (MASP) + extraction with MeOH 80%; (C) microwave-assisted extraction (MAE) with a solvent/raw material ratio of 1:20 or 1:4. Created in BioRender. Fernández hernández, N. (2026) https://BioRender.com/t7a20pj (accessed on 5 March 2026).

Polar extracts were prepared using different extraction methods (Figure 1). Following extraction, the samples were analyzed by HPLC-DAD at 280 nm to quantify the total phenolic content (TPC).

The first extraction method (Figure 1, A), here referred to as conventional extraction (CE), was based on a previous study by Silva et al. [9]. To evaluate potential improvements, a microwave-assisted pre-treatment step was incorporated prior to the conventional extraction procedure (MASP + CE; Figure 1, B). The mass yields were comparable between CE and MASP + CE (4.14 ± 0.41 and 4.14 ± 0.18% DWB, respectively). Total phenolic content values also showed no significant differences with the inclusion of MASP, indicating that this pre-treatment did not enhance the extraction of phenolic compounds from the matrix.

As part of a greener approach, microwave-assisted extraction (MAE) was also evaluated as an alternative to conventional extraction (Figure 1, C), aiming to achieve higher efficiency in a shorter time. Environmentally friendly solvents such as ethanol (EtOH) and water (H_2_O), and their hydroalcoholic mixtures at 50% (E50) and 80% (E80) were used. For comparison with conventional methods, methanol (MeOH) and its mixtures at 50% (M50) and at 80% (M80) were also used. Two sample/solvent ratios were tested by varying the amount of olive stones (1 or 5 g), while keeping the solvent volume constant at 20 mL.

**Figure 2:**
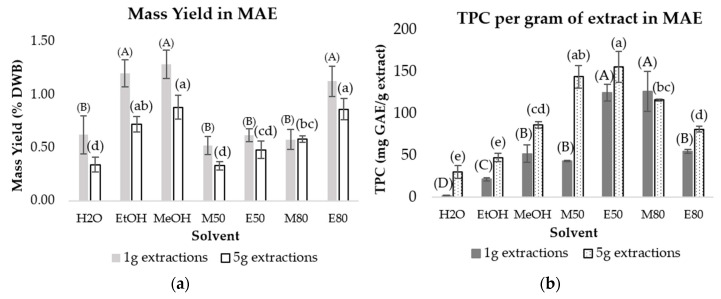
(**a**) Average mass yield obtained after microwave assisted extractions (MAE) of polar compounds using 1 and 5 g of olive stones; (**b**) Total phenolic content (TPC) obtained per g of olive stones extract using MAE (1 g or 5 g of raw material). Solvents used: water (H_2_O), ethanol (EtOH), methanol (MeOH), and aqueous MeOH (M50 and M80) and EtOH mixtures (E50 and E80). DWB—dry weight basis, GAE—gallic acid equivalents; Different letters indicate significantly different means, as determined by an ANOVA test (*p* < 0.05).

The 5 g microwave-assisted extracts contained lower amounts of non-phenolic compounds and higher concentrations of phenolic compounds, as reflected by their lower mass yields (Figure 2a) and higher total phenolic contents per gram of extract (TPC/g) (Figure 2b). Notably, MAE using aqueous MeOH and EtOH mixtures resulted in higher TPC/g extract compared to extractions using pure EtOH or MeOH as solvents, particularly when using 5 g of olive stones (Figure 2b).

**Figure 3:**
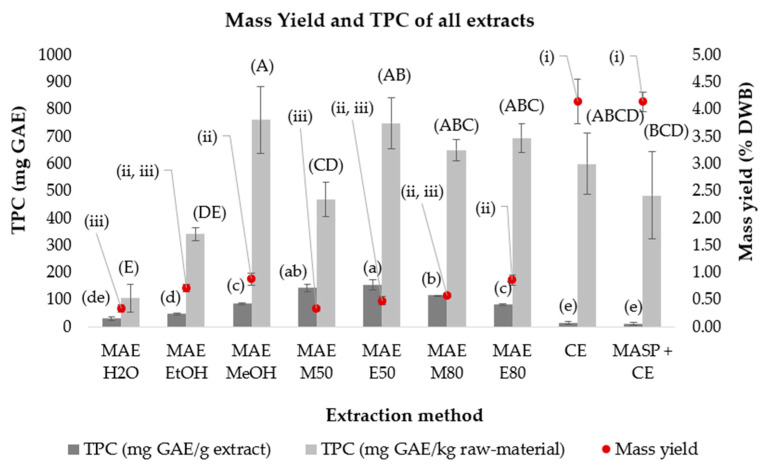
Mass yield and total phenolic content (TPC) in olive stones obtained after microwave assisted extraction (MAE), conventional extraction (CE), and microwave assisted sample pretreatment followed by conventional extraction (MASP + CE); DWB—dry weight basis. Solvents used: water (H_2_O), ethanol (EtOH), methanol (MeOH), and aqueous MeOH (M50 and M80) and EtOH mixtures (E50 and E80) (%*v*/*v*). GAE—gallic acid equivalents. Different letters in parentheses within each assay indicate significantly different means, as determined by an ANOVA test (*p* < 0.05). Uppercase letters refer to TPC (mg GAE/kg raw-material), lowercase letters refer to the TPC (mg GAE/g extract) and Roman numerals (i–iii) for mass yield.

The 5g MAE M80 extract showed similar values of TPC extracted per kg of olive stones in comparison to CE (Figure 3), indicating that the extraction efficiency of MAE and CE was similar when the same solvent was used. Moreover, the higher mass yield and lower TPC/g of extract obtained for CE, when compared to MAE, suggested that CE method extracts a higher amount of non-phenolic compounds.

The difference in mass yields observed between the 1 and 5 g MAE extractions suggests that using 5 g in the extraction, the solvent might saturate, thus preventing the extraction of more compounds and leading to a lower mass yield.

The 5 g MAE method also yielded similar TPC per kg of olive stone when compared to CE. Additionally, its remarkably higher TPC value per g of extract and lower mass yields indicate that MAE is more efficient for selective extraction of phenolic compounds.

The higher TPC values per g of extract obtained with aqueous mixtures of MeOH and EtOH in MAE are in accordance with those reported by Osei et al. [38]. Furthermore, TPC values of 4.37 to 8.73 [38], 1.0 to 2.4 [39] and 3.56 to 11.50 [21] mg GAE/g of olive stone (DW), obtained by the Folin-Ciocalteu method, have been reported in the literature. This method is highly susceptible to matrix effects that likely contribute to the considerable variability among reported values [40]. In addition, the different extraction methods as well as other parameters such as the different olive cultivars and the maturity of the fruits can also influence TPC values [41].

The 5g extracts obtained by CE and MAE were selected, based on their higher TPC/g values, for further compound identification using HPLC-DAD-MS/MS and evaluation of antioxidant activity.

### 2.3. Phenolic Profile of Extracts Identified by HPLC-DAD-MS/MS

High performance liquid chromatography coupled with diode array and mass spectrometry detectors analyses were conducted to identify the major phenolic compounds present in the studied extracts. The MS chromatograms were acquired using full scan and multiple reaction monitoring (MRM) modes, which allowed for better selectivity and sensitivity. The MRM analytical parameters were optimized using 52 commercial standards, comprising small phenolic compounds, flavonoids and flavonoids glycosides, phenylpropanoid glycosides, and secoiridoids. These compounds have been reported to occur in olive tree leaves, olive-derived by-products and olive oil [42,43,44,45]. Some non-phenolic compounds were also searched, including citric, quinic and succinic acids.

Although numerous peaks were detected by DAD, particularly at 280 nm wavelength, a total of 35 phenolic compounds were unequivocally identified by comparison of their retention time (Rt) and *m*/*z* values with respective authentic standards (Table 1).

The highest number of phenolic compounds were identified in microwave assisted extracts using hydroalcoholic mixtures, particularly in E50 (29 compounds). E80 showed slightly reduced diversity compared to E50, missing cyanidin-3-O-rutinoside, genistein, phloretin and some small phenolic compounds (gallic, vanillic and syringic acids, and vanillin). A comparable number of compounds were identified in MeOH, M50 and M80 extracts, despite the slightly different chemical profile. In the CE extract it was possible to identify 21 compounds, similarly to the M80 MAE extract. Comparing the results obtained for CE and MASP + CE (12 compounds), it was observed that in this case the use of microwave pretreatment was disadvantageous, particularly for the extraction of some compounds such as flavonoid glycosides (luteolin-7-glucoside, cyanidin 3-o-rutinoside, hesperidin and rutin), and also catechin gallate, phloretin, protocatechuic, 3,4,5-trimethoxycinnamic, ferulic, and p-coumaric acids. Conversely, some of their aglycones (e.g., luteolin and quercetin) were present in CE extracts, suggesting that the microwave-assisted pre-treatment step followed by the conventional extraction for 45 min can promote the hydrolysis of glycosidic bonds [46]. The absence of several flavonoids and phenolic acids in the MASP-treated extract supports the need for careful optimization when combining pretreatment steps with conventional protocols.

Some compounds were identified in all the extracts, including flavonoids (luteolin, apigenin, quercetin, and naringenin), and syringaldehyde, which appear to be highly extractable regardless of solvent polarity and extraction method. Conversely, compounds such as kaempferol, cyanidin 3-O-rutinoside, catechin gallate, and some small phenolic compounds, such as gallic, syringic, chlorogenic, vanillic, and 3,4,5-trimethoxycinnamic acids, were detected only in specific extracts. The phenylpropanoid glycosides verbascoside and salidroside, as well as the secoiridoids nuezhenide and oleuropein, were also found in all the extracts analyzed. Tyrosol was not detected in any of the extracts, even though hydroxytyrosol was identified in all MAE extracts. However, hydroxytyrosol was not found in the extracts obtained by CE or MASP + CE. Both hydroxytyrosol and tyrosol are common phenolic compounds in virgin and extra virgin olive oil and olives, and they have also been identified in olive leaves and other olive byproducts [47]. Many of the compounds identified in this work have been previously reported in olive seed and stones, with emphasis on nuezhenide, oleuropein, and hydroxytyrosol [9,20,21,22,23,24,25,47,48,49,50,51,52,53].

### 2.4. Antioxidant Activity of the Polar Compound Extracts

**Figure 4:**
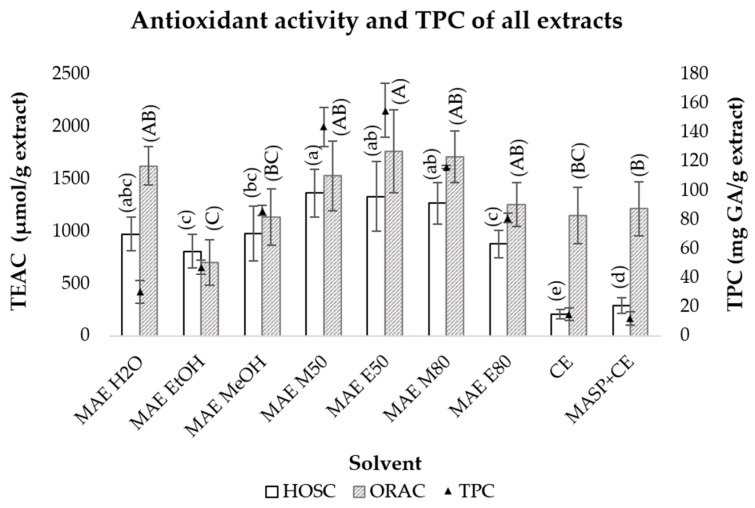
Antioxidant activity of olive stones extracts was determined using the ORAC and HOSC assays, as well as the total phenolic content (TPC). Olive stone extracts were obtained from microwave assisted extraction (MAE), conventional extraction (CE), and microwave assisted sample pretreatment followed by conventional extraction (MASP + CE); Solvents used: water (H_2_O), ethanol (EtOH), methanol (MeOH), and aqueous MeOH (M50 and M80) and EtOH mixtures (E50 and E80) (%*v*/*v*). Different letters in parentheses within each assay indicate significantly different means, as determined by an ANOVA test (*p* < 0.05). Bars sharing the same letter are not significantly different within their respective assay. Uppercase letters refer to the ORAC TEAC values, and lowercase letters refer to the HOSC TEAC values.

The antioxidant activity was assessed using the ORAC and the HOSC assays (Figure 4). The ORAC assay evaluates the ability of the extract to react with peroxyl radicals (ROO^•^), whereas the HOSC assay determines its capacity to scavenge hydroxyl radicals (^•^OH), both of which are biologically relevant species implicated in oxidative stress.

The antioxidant activity values obtained from both assays were comparable across most MAE extracts except for the H_2_O extract, which exhibited higher values in the ORAC assay (Figure 4). A positive correlation was observed between the antioxidant activity measured using the HOSC assay and the total phenolic content (TPC) values (R^2^ = 0.8041), suggesting that the phenolic compounds are likely the main contributors to the observed antioxidant ability. A positive correlation (R^2^ = 0.8366) was also found between the values determined with the ORAC assay and TPC values of MAE extracts, except for the water extract, which showed a lower TPC/g of extract and high antioxidant activity. This suggests the presence of non-phenolic compounds that potentially scavenge peroxyl radicals. Similar ORAC values were also obtained for CE and M80 MAE extracts, despite lower TPC values in CE extracts. Previous studies have reported the presence of polysaccharides in olive stone, which are also known to exhibit antioxidant activity [54].

The values of HOSC per gram of extract obtained for MAE were significantly higher than those obtained for CE, but the same did not happen for the ORAC assay, as the values obtained were very similar between both methods. Overall, the E50, M50, and M80 MAE extracts presented higher antioxidant capacity (ORAC and HOSC values), and higher TPC per g of extract.

Comparable ORAC values have been previously reported in the literature. However, in this study, higher HOSC values were observed [55]. Similar ORAC values were also reported for other olive by-products, such as leaves [56] and pomace [57], highlighting the antioxidant potential of olive stones. These findings suggest that olive stones may represent a promising source of bioactive compounds with health-promoting properties.

### 2.5. Citotoxicity and Antiproliferative Activity

The higher antioxidant activity values and higher total phenolic content per g of extract determined for E50 and M80 MAE extracts highlight their potential for further bioactivity assays.

**Figure 5:**
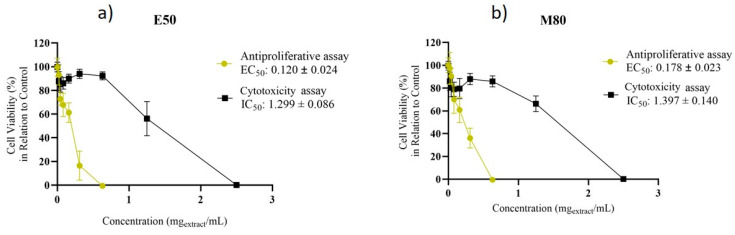
(**a**) Cytotoxicity and antiproliferative activity of E50 MAE extract on Caco-2 and HT-29 cells, respectively; (**b**) Cytotoxicity and antiproliferative activity of M80 MAE extract on Caco-2 and HT-29 cells, respectively. Results presented as mean ± SD (*n* = 4).

The cytotoxicity of E50 and M80 extracts, reconstituted in ethanol, was evaluated in Caco-2 cells, a widely used model of the human intestinal epithelium. Both extracts showed a similar cytotoxicity towards Caco-2 cells (Figure 5). Moreover, concentrations up to 0.63 mg extract/mL (73.14 and 54.11 mg olive stone/mL for E50 and M80, respectively) did not result in significant toxicity, as cell viability remained above 70% relative to controls, in accordance with ISO 10993-5:2009 [58].

The antiproliferative activity of E50 and M80 extracts was also assessed by using the HT-29 cell line as a model for human colorectal adenocarcinoma. None of the concentrations tested (0.02–0.63 mg/mL) induced cytotoxicity in Caco-2 cells according to ISO 10993-5:2009 [54]. Both E50 and M80 extracts resulted in a total loss of HT-29 cell viability at concentrations of 0.63 mg/mL or higher (Figure 5). The EC_50_ and TPC values obtained for the E50 extract (0.12 ± 0.024 and 155 ± 19 mg GAE/g extract, respectively) were significantly different (*p* < 0.05) from those of M80 (0.178 ± 0.023 and 116.00 ± 1.04 mg GAE/g extract, respectively). These results indicate that the E50 extract, which showed a higher antiproliferative effect against HT-29 cells also showed higher total phenolic content and antioxidant capacity. These results are in agreement with the literature demonstrating that phenolic compounds, primarily responsible for antioxidant activity, exhibit potential antiproliferative effects in colorectal cancer by fighting oxidative stress, inhibiting proliferation of malignant cells, inducing apoptosis, and modulating signaling pathways central to disease development [32,59]

Colorectal cancer is the third most common cancer, accounting for an estimated 1.9 million new cases worldwide. Moreover, the disease causes a massive morbidity and mortality, with more than 900,000 deaths occurring worldwide, making it the second leading cause of cancer-related death. Incidence rates were highest in Europe and in Australia and New Zealand, while mortality rates were highest in Eastern Europe [60]. Since polyphenolic compounds have high antioxidant capacity and potential anti-inflammatory and anti-proliferative effects, they can help mitigate oxidative stress and be advantageous for the prevention of disorders like colorectal cancer, which is largely attributable to modifiable environmental and lifestyle factors such as diet, tobacco, and alcohol, among others [61,62]. Preclinical research has demonstrated that polyphenols possess inherent anti-colon-cancer properties and exhibit multifactorial mechanisms of action, involving modulation of intracellular signaling pathways, regulation of gene transcription, and attenuation of cellular oxidative stress [63]. The effects of some phenolic compounds identified in extracts on HT-29 cells were previously reported. Oleuropein has been proven to have an antiproliferative effect and induce apoptosis by activating the p53 pathway [63,64]. Luteolin was found to suppress cell proliferation, exhibiting a dose-dependent effect [65,66,67], and genistein inhibited cell proliferation and induced apoptosis by activating the executioner caspase-3 [68].

Overall, results showed that the E50 extract presented antiproliferative potential against colorectal cancer cell lines and could be promising for further studies, such as the evaluation of its combinatorial effect with chemotherapeutic drugs.

## 3. Materials and Methods

### 3.1. Sample

Ground olive stones from different cultivars, obtained after olive oil extraction, were provided by Sovena Portugal—Consumer Goods, S.A (Algés, Portugal). The sample was delivered to iBET’s facilities in the summer of 2022 and dried at 100 °C in a drying chamber (WTC ED115, Binder GmbH, Tuttlingen, Germany). The sample was further ground in a ball mill (Retsch MM400, Haan, Germany) at a frequency of 30 Hz for 30 s.

### 3.2. Solvents and Reagents

n-Hexane (Carlo Erba, Cornaredo, Italy), chloroform (Carlo Erba, Cornaredo, Italy), methanol (Fisher Chemical, Pittsburgh, PA, USA), and isooctane (Honeywell Riedel-de-Haen, Seelze, Germany) were used for lipids extraction. Sodium hydroxide (Fisher Chemical, Loughborough, UK), a 14% boron trifluoride in methanol solution (Sigma Aldrich, Munich, Germany), and butylated hydroxytoluene (BHT, VWR International, Leuven, Belgium) were used for FA methylation. Nonadecanoic acid (Sigma Aldrich, Munich, Germany) was used as internal standard. Sodium metabisulfite (98–100.5%, Sigma Aldrich, Munich, Germany), methanol (≥ 99.8%, Fisher Chemical, Loughborough, UK), Milli-Q H2O and ethanol (≥99.8%, Honeywell Riedel-de-Haen, Seelze, German) were used for polar extractions. For HPLC analysis Milli-Q water and HPLC-grade solvents were used. The standards gallic acid, cyanidin 3-*o*-rutinoside, luteolin, apigenin, 3,4,5-trimethoxycinnamic acid, vanillin, hesperidin, gallocathechin gallate, luteolin 7-glucoside, catechin gallate, chlorogenic acid, isorhamnetin, quercetin, catechin, phloretin, ferulic acid, citric acid, caffeic acid, vanillic acid, *o*-coumaric acid, cinnamic acid, *m*-hydroxybenzoic acid, succinic acid, epicatechin, pelargonin, myricetin, kaempferol, naringenin, syringaldehyde, benzoic acid, spiraeoside, rutin, epigallocatechin gallate, quercitrin, epicatechin gallate, phlorizin, epigallocatechin, ellagic acid, epicatechin, genistein, syringic acid, quinic acid, p-coumaric acid, m-coumaric acid, protocatechuic acid, p-hydroxybenzoic acid, and salidroside were obtained from Sigma Aldrich, Munich, Germany; nüzhenide, oleuropein, tyrosol, hydroxytyrosol and verbascoside were obtained from Extrasynthese, Genay, France; oleuropein aglycone was obtained from Biosynth, Staad, Switzerland.

For antioxidant activity assays, fluorescein sodium salt, sodium chloride, potassium phosphate monobasic, sodium phosphate dibasic dihydrate, 2,2′-Azobis(2-amidinopropane)dihydrochloride (AAPH), 6-hydroxy-2,5,7,8-tetramethylchroman-2-carboxylic acid (Trolox), sodium phosphate monobasic monohydrate, and iron chloride were purchased from Sigma Aldrich (Munich, Germany). Potassium chloride was obtained from Alfa Aesar (Ward Hill, MA, USA), H_2_O_2_ 30% from Carlo Erba (Cornaredo, Italy), and acetone from Labchem (Zelienople, PA, USA).

For the culture mediums, 0.25% Trypsin-ethylenediaminetetraacetic acid solution (Trypsin-EDTA 1X, Gibco, Thermo Fisher Scientific, Waltham, MA, USA), heat inactivated fetal bovine serum (FBS, Biowest, Nuaillé, France), Minimum Essential Medium Non-Essential Amino Acids solution (MEM NEAA 100X, Gibco, USA), penicillin streptomycin (PS, Gibco, Thermo Fisher Scientific, Waltham, MA, USA), Dulbecco’s Modified Eagle Medium (DMEM 1X, Corning, New York, NY, USA) and Roswell Park Memorial Institute Medium (RPMI 1640, Biowest, France) were used. For the cell viability assay, PrestoBlue cell viability reagent (Invitrogen, Carlsbad, CA, USA) was used along with phosphate buffered saline (PBS, pH 7.4) and trypan blue stain (0.4%) from Gibco, Thermo Fisher Scientific (Waltham, MA, USA), the later for cell counting.

### 3.3. Lipid Extraction

The Soxhlet and Folch methods were used for lipid extraction. The Soxhlet extraction was performed according to the procedures described by Moussaoui et al. [69] and Maestri et al. [22]. Briefly, 300 mL of *n*-hexane was added to 15 g of raw-material, and the extraction process occurred for 6 h.

The Folch method was carried out as previously described by Alves et al., with some modifications [70,71]. First, 9 g of raw material was added to a flask, along with 180 mL of CHCl_3_/MeOH (2:1) mixture and stirred for 30 min at room temperature. The solvent was then filtered into a separatory funnel, and 36 mL of distilled water was added to induce phase separation. The organic phase was collected, and 90 mL of the CHCl_3_/MeOH (2:1) mixture, along with 18 mL of water were added to the aqueous layer in the funnel, allowing for another phase separation. The organic layer was collected to ensure the full recovery of the lipid fraction.

After each extraction, the solvent was evaporated in a rotary evaporator at 40 °C, and the dry extract was stored at −20 °C until further analysis.

### 3.4. Fatty Acids Derivatization

The derivatization process into fatty acid methyl esters (FAMEs) was conducted according to a reported procedure [72]. First, the dry sample was reconstituted in 6 mL of CHCl_3_ and divided into three tubes, adding 500 μL of nonadecanoic acid (internal standard,1 mg/mL in CHCl_3_ solution). Saponification reaction carried out at 60 °C after the addition of 1 mL of a 0.5 M methanolic NaOH. After cooling at room temperature, 1 mL of a 14% methanolic solution of BF_3_ was added to induce the methylation reaction, with continuous stirring for 30 min, at a temperature of 60 °C. Subsequently, 1 mL of a solution containing 5 μg/mL BHT in isooctane was added to each tube, and the mixture was vortexed for liquid–liquid separation. Finally, 500 μL of the upper phase from each tube was collected for further analysis, and stored at −20 °C, protected from light.

### 3.5. Fatty Acid Quantification (GC-FID)

The fatty acid (FA) absolute quantification was performed by gas chromatography coupled to a flame ionization detector (GC-FID) according to a procedure previously reported [73]. Analyses were carried out on a Trace GC 2000 system equipped with a flame ionization detector (FID) operating at 280 °C (Thermo Scientific, Waltham, MA, USA) and a DB-23 fused silica column (60 m × 0.25 mm ID × 0.25 μm film thickness, Agilent J&W, Folsom, CA, USA). The injection volume was 2 μL, and the injector’s temperature was set to 220 °C. The oven temperature was increased from 70 °C to 195 °C at 5 °C/min, held at 195 °C for 30 min, then increased to 220 °C at the same rate and held for 60 min. Helium was used as the mobile phase at a constant pressure of 70 kPa. Nonadecanoic acid was used as an internal standard. The FAMEs were identified by comparing their relative retention times with those of a standard mixture of 52 FAMEs (Nu-Chek-Prep Inc., Elysian, MN, USA). The absolute quantification of each FA in the extracts was performed according to Equation (1), where IS corresponds to internal standard nonadecanoic acid.

(1) $$FA\;concentration\;(\mu g/mg\;extract)\;=\frac{FAME\;peak\;area}{IS\;peak\;area}\times \frac{IS\;weight\;(\mu g)}{extract\;weight\;(mg)}$$

### 3.6. Polar Compounds Extraction

#### 3.6.1. Solid-Liquid Conventional Extraction (CE)

A conventional approach to the extraction of phenolic compounds from olive stones was based on previous research by Silva et al. (2006) [9] with some modifications. Briefly, 10 mL of a sodium metabisulfite 2% solution were added to 5 g of sample with 50 mL of an aqueous MeOH 80% (%*v*/*v*) solution. The mixture was stirred for 15 min at room temperature and the process was repeated twice with the remaining solid residue.

#### 3.6.2. Microwave Assisted Sample Pretreatment (MASP)

A microwave-assisted sample pretreatment (MASP) was applied prior to conventional extraction (CE) to enhance extraction efficiency. Into a quartz reactor tube, 5 g of raw material were added, along with approximately 3 mL of MeOH 80% (%V). The tube was then placed in a microwave system (Discover SP, CEM Corporation, Matthews, NC, USA) and the sample was irradiated at a temperature of 60 °C and at maximum power (300 W) during 5 min, after a ramping time of another 5 min. Next, the conventional methodology was conducted using the treated sample.

#### 3.6.3. Microwave Assisted Extraction (MAE)

Seven solvents were used for MAE, namely, H_2_O, EtOH, MeOH, and aqueous solutions of EtOH or MeOH at 50% and 80% (*v*/*v*). Samples (1 or 5 g) were added to a quartz reactor tube, along with 20 mL of solvent. The mixture was placed in the microwave equipment (Discover SP, CEM Corporation, Matthews, NC, USA ) and irradiated at a temperature of 60 °C and at maximum power (300 W) for 5 min, after a ramping time of another 5 min and 30 s of pre-stirring. After the extraction, the solvents were partially evaporated in a rotary evaporator at 40 °C to a final volume of 25 mL. The remaining extract volume and the extracts from MAE were then transferred into 50 mL centrifuge tubes (VWR, Radnor, PA, USA) and evaporated to dryness in a centrifugal vacuum concentrator (CentriVap, Labconco, Kansas City, MO, USA) with a cold trap (−84 °C CentriVap Cold Trap, LabConco, Kansas City, MO, USA), to facilitate storage. The dried extracts were stored at −20 °C until analysis.

### 3.7. Determination of Total Phenolic Content (TPC) by HPLC-DAD

The dried polar extracts were reconstituted with their respective extraction solvents, filtered through a 0.22 μm polyvinylidene difluoride (PVDF) membrane (Whatman, Leeds, UK) and diluted with EtOH 50% to concentrations of around 4 mg/mL. The TPC was determined by HPLC-DAD using a method previously described by Weng et al. with some modifications [74]. The system used was a Vanquish HPLC system (Thermo Fischer Scientific, Waltham, MA, USA) and it consisted of a pump, coupled to a diode array detector (HPLC-DAD), and an autosampler. The procedure was carried out using a RP Luna C18 (5 μm, 250 × 4 mm, Phenomenex, Torrance, CA, USA) column, operating at 35 °C. The injection volume was 10 μL and the flow rate was 0.6 mL/min. The mobile phase consisted of a mixture of two eluents, A: 0.5% HCOOH in Milli-Q^®^ water and B: 90% ACN and 0.5% HCOOH in aqueous solution. The following gradient of eluents was used: 5.6% B over 5 min, 5.6–20% B from 5 to 15 min, 20–40% B from 15 to 22 min, held isocratic at 40% B for 10 min, 40–100% B from 32 to 45 min, held isocratic at 100% B for 5 min, followed by an equilibration step of 10 min. TPC values were expressed in gallic acid equivalents (GAE) using solutions prepared from the commercial standard, at 280 nm, at concentrations ranging from of 1 to 100 μg/mL.

### 3.8. Identification of Phenolic Compounds by HPLC-DAD-MS/MS

Phenolic compounds were identified by HPLC-DAD-MS/MS, using a Waters Alliance 2695 (Waters, Wexford, Ireland) HPLC system equipped with a quaternary pump, a solvent degasser, an autosampler and a column oven, coupled to a Waters 996 DAD (Waters, Wexford, Ireland). Tandem mass spectrometry (MS/MS) detection was performed using a Micromass Quattro Micro triple quadrupole (Waters, Wexford, Ireland) with an electrospray ionization source, operated at 120 °C. A capillary voltage of 2.5 kV and a source voltage of 30 V were applied in scan detection mode. The DAD was set to scan the wavelength absorption from 210 to 700 nm. The flow rate was 0.3 mL/min, and 20 μL of each extract at 10 mg/mL was injected into the column. The mobile phase consisted of a mixture of two eluents, A: 0.1% HCOOH in Milli-Q^®^ water and B: acetonitrile. The following gradient was used: 5% B over 10 min, 5–18% B from 10 to 30 min, 18–36% B from 30 to 44 min, held isocratic 36% B for 20 min, 36–90% B from 64 to 90 min, held isocratic at 90% B for 10 min, followed by an equilibration step of 20 min. Individual solutions of each standard were analyzed in order to determine the optimal cone voltage and collision energy for multiple reaction monitoring mode (MRM) (Supplementary Table S3). High-purity nitrogen was used as the nebulizing and drying gas and ultra-high purity argon was used as the collision gas. MassLynx software v.4.1. was used to control the system, for data acquisition and processing.

### 3.9. Antioxidant Capacity Assays

#### 3.9.1. Oxygen Radical Absorbance Capacity (ORAC) Assay

This assay was an adaptation of the method previously described by Huang et al. (2002) [75]. Samples and Trolox solutions were prepared with phosphate buffered saline (PBS, 75 mM, pH 7.4). Trolox standard solutions ranging from 5 to 40 μM were prepared. The 60 inner wells of a 96 well black microplate were filled with 25 μL of PBS, Trolox standards or samples and 150 μL of a fluorescein solution (3 × 10^−4^ mM). The microplate was incubated for 10 min at 37 °C in a microplate fluorimeter (FL800 microplate reader, BioTek Instruments, Winooski, VT, USA) before 25 μL of an AAPH solution (153 mM) were added to the wells. Fluorescence measurements were performed using an excitation wavelength of 485 ± 20 nm and an emission wavelength of 528 ± 20 nm. The kinetic reaction was followed for 40 min and the data were analyzed by the BioTEK Gen 5 software (v 3.12.08). For every replicate of each extract, three dilutions were performed and each diluted sample was analyzed in triplicate.

#### 3.9.2. Hydroxyl Radical Scavenging Capacity (HOSC) Assay

This assay was an adaptation of a method previously described by Moore et al. (2006) [76], with the samples and the Trolox standards being diluted with sodium phosphate buffer (SPB, 75 mM, pH 7.4), which was also used as a blank. Trolox standards with concentrations ranging from 5 to 30 μM were prepared. Before the reading, the plate reader incubation chamber was set to 37 °C. To the 60 inner wells of a black microplate, 30 μL of PBS, Trolox standards or sample (according to the plate layout) were added along with 170 μL of a FL solution (9.96 × 10^−8^ M), 40 μL of a 0.20 M solution of H_2_O_2_ and 60 μL of a 3.42 mM solution of FeCl_3_. The fluorescence was then read in a fluorimeter (FL800 Bio-Tek Instruments, Winooski, VT, USA) using an excitation wavelength of 485 ± 20 nm and an emission wavelength of 528 ± 20 nm. The kinetic reaction was followed for 60 min, and the data were analyzed using BioTEK Gen 5 software (v. 3.12.08). For every replicate of each extract, four dilutions were performed, and each diluted sample was analyzed in triplicate. Each extraction solvent was diluted and assayed to account for its effect on the final result.

### 3.10. Cell-Based Assays

Dried extracts were reconstituted in EtOH and filtered through sterile polyethersulfone 0.22 μm filters (Filter-Bio, Nantong, China). Each extract was diluted in the adequate assay medium, with the most concentrated containing only 10% EtOH, to ensure compatibility with the cell lines.

#### 3.10.1. Cell Cultures

The human cell lines used were Caco-2 (HTB-37™), a human epithelial cell model of the intestinal barrier, derived from a colorectal adenocarcinoma, and the HT-29 (HTB-38™) human colon adenocarcinoma cell line, both supplied by American Type Culture Collection (ATCC, Manassas, VA, USA). Caco-2 cells were grown in DMEM 1X supplemented with 10% (%V) heat inactivated fetal bovine serum, 1% Minimum Essential Medium Non-Essential Amino Acids solution and 1% penicillin streptomycin. HT-29 cells were cultured in RPMI 1640 medium, supplemented with 10% FBS. Stock cells were incubated at 37 °C with 5% CO_2_ in a humidified atmosphere inside 75 cm^2^ culture flasks, as monolayers. Both cytotoxicity and antiproliferative activity assays were performed according to a previous report by Oliveira-Alves et al. [77].

#### 3.10.2. Cytotoxicity and Antiproliferative Assays

For the cytotoxicity assay, undifferentiated Caco-2 cells were used [78,79]. After reaching confluence, these cells were seeded into the 60 inner wells of 96-well microplates, at a density of 2 × 10^4^ cells/well and allowed to grow for 7 days. The assay medium consisted of DMEM supplemented with 0.5% FBS and 1% NEAA, and it was renewed every 48 h. After a week, the cells were incubated with 100 μL of assay medium (control) and different concentrations of the extracts ranging from 2.5–0.02 mg/mL. After 72 h, the PrestoBlue cell viability assay was performed according to the manufacturer’s instructions in a fluorimeter (FL800 microplate reader, BioTek Instruments, Winooski, VT, USA).

HT-29 cells were used for antiproliferative activity assays. After reaching confluence, these cells were seeded into the 60 inner wells of 96-well microplates at a density of 1 × 10^4^ cells/well and allowed to grow for 7 days. The assay medium consisted of DMEM supplemented with 0.5% FBS and 1% NEAA, and it was renewed every 48 h. After a week, the cells were incubated with 100 μL of assay medium (considered the control) and with different concentrations of the extracts ranging from 2.5 to 0.02 mg/mL. After 72 h, the PrestoBlue cell viability assay was performed according to manufacturer’s instructions in a FL800 microplate reader (BioTek Instruments, Winooski, VT, USA). 

### 3.11. Statistical Analysis

All results are expressed as “mean ± standard deviation”. Statistical analysis was performed using R 4.4.1. Software, except for cell-based assays, which were processed in GraphPad 10.2.3. The EC_50_ (half-maximal effective concentration) was determined by nonlinear regression with a 95% confidence interval. One-way analysis of variance (ANOVA) was performed, followed by Tukey’s Honest Significant Difference test for multiple comparisons; however, the results are limited by the small sample size [80]. A *p*-value lower than 0.05 was considered statistically significant for all cases.

## 4. Conclusions

To contribute to the sustainable valorization of olive stones, this study explored the preparation of several extracts by microwave-assisted extraction (MAE) using ethanol and water, and their hydroalcoholic mixtures as an alternative, environmentally friendly method. Methanol and its aqueous mixtures were also used in MAE to compare the chemical profile of the MeOH 80% extract with that obtained by conventional extraction (CE), as reported in the literature. The resulting extracts were subsequently characterized and compared with respect to their chemical composition and bioactive properties. Furthermore, the fatty acid profile of olive stones was also compared using the Soxhlet and Folch methods, widely used for lipid extraction. The selection of an appropriate solvent is the most critical factor in the efficient extraction of lipids. Soxhlet uses a hot non-polar solvent to extract mainly neutral lipids, while Folch uses a cold chloroform–methanol mixture to disrupt the protein–lipid complexes and extract a broader range of lipids [81].

The Soxhlet extraction showed higher efficiency in recovering larger quantities of the major fatty acids (FA)—oleic, palmitic, and linoleic acids—as well as a wider range of these compounds. As expected, the Soxhlet extract contained higher concentrations of unsaturated fatty acids, both monounsaturated and polyunsaturated, reaching 363.03 μg/mg and 65.39 μg/mg, respectively. In contrast, the Folch extract yielded lower amounts, with 218.65 μg/mg of monounsaturated and 29.54 μg/mg of polyunsaturated fatty acids. Future studies should focus on the application of modern and environmentally friendly techniques, such as supercritical CO_2_ extraction, to potentially enhance efficiency and sustainability.

Microwave-assisted pre-treatment (MASP) prior to CE did not significantly improve the extraction of phenolic compounds. In contrast, MAE—particularly when using lower solvent-to-sample ratios and aqueous solvent mixtures—showed better efficiency. It enhanced the selectivity for phenolic compounds, yielding higher TPC per gram of extract and a reduced extract mass, minimized the co-extraction of non-phenolic compounds, and maintained comparable TPC values per kilogram of raw material.

Microwave pretreatment (MASP + CE) reduced the recovery of several flavonoid glycosides and phenolic acids. Some compounds, like luteolin, apigenin, quercetin, syringaldehyde, verbascoside, salidroside, nüzhenide and oleuropein, were identified in all extracts. Hydroxytyrosol was exclusive to MAE extracts, and tyrosol was not detected in all the samples. These results underscore the need to optimize extraction protocols to preserve compound diversity.

The MAE extracts showed higher hydroxyl radical scavenging capacity (HOSC) and similar oxygen radical absorbance capacity (ORAC) values when compared to CE. Moreover, a correlation analysis between TPC and ORAC values suggests that CE extracts and water MAE extracts may contain non-phenolic compounds with enhanced peroxyl radical-scavenging activity.

Both E50 and M80 extracts showed comparable cytotoxic effects, with E50 exhibiting higher antiproliferative activity. A concentration of 73.14 mg olive stone/mL could be a starting point for future studies.

The results presented herein highlight the potential of olive stone extracts for future high-value applications in the pharmaceutical and nutraceutical sectors, extending their use beyond traditional energy generation via combustion. Despite their promise, the use of olive stones is limited by factors such as compositional variability, the presence of a cellulose matrix that restricts extraction efficiency, and challenges associated with valorizing a byproduct at an industrial scale. These constraints highlight the need for standardized pretreatment methods and improved process control to ensure consistent chemical profiles and reproducible bioactivity.

This study, although exploratory, establishes a solid foundation for further research, including the quantitative analysis of the identified bioactive compounds and the characterization of the antiproliferative effect of isolated compounds to further elucidate mechanisms of action and assess combinatorial effects with anticancer drugs. Future research should focus on optimizing green extraction technologies and exploring targeted bioactivities to better support the integration of olive stone-derived compounds into the referred applications. Additionally, studying scalable processing approaches and advanced purification strategies will be essential to fully harness the high-value potential of these residues.

## Figures and Tables

**Table 1:** Phenolic compounds identified in olive stone extracts by HPLC-DAD-MS/MS. MAE—microwave assisted extraction; CE—conventional extraction; MASP + CE—microwave assisted sample pretreatment + conventional extraction; ✔—identified in the extract. (+) compounds detected in positive ionization mode; (−) compounds detected in negative ionization mode.

				MAE								
	Rt (min)	*m*/*z*	MS/MS Ions	H_2_O	EtOH	E50	E80	MeOH	M50	M80	CE	MASP + CE
**Flavonoids**												
Gallocatechin gallate	43.28	457 (−)				✔	✔		✔			
Cyanidin 3-O-rutinoside	44.72	595 (+)	287, 449			✔					✔	
Rutin	46.46	609 (−)	271, 300	✔	✔	✔	✔	✔	✔	✔	✔	
Catechin gallate	47.67	441 (−)	169, 289			✔	✔				✔	
Luteolin 7-glucoside	47.85	447 (−)	284, 285	✔	✔	✔	✔	✔	✔	✔	✔	
Hesperidin	49.76	609 (−)	228, 301		✔		✔	✔	✔		✔	
Luteolin	58.23	285 (−)	107, 133	✔	✔	✔	✔	✔	✔	✔	✔	✔
Quercetin	58.36	301 (−)	151, 179		✔	✔	✔	✔	✔	✔	✔	✔
Phloretin	62.56	273 (−)	123, 167			✔		✔	✔	✔	✔	
Naringenin	63.72	273 (+)	147, 153	✔	✔	✔	✔	✔	✔	✔	✔	✔
Genistein (isoflavonoid)	65.54	269 (−)	63, 133		✔	✔		✔				
Apigenin	66.26	271 (+)	119, 153	✔	✔	✔	✔	✔	✔	✔	✔	✔
Kaempferol	67.44	287 (+)	121, 153									✔
Isorhamnetin	69.85	315 (−)	151, 300			✔	✔				✔	✔
**Small phenolic compounds**												
Gallic acid	15.14	169 (−)	79, 125			✔						
Protocatechuic acid	26.93	153 (−)	81, 109	✔	✔	✔	✔	✔	✔	✔	✔	
Hydroxytyrosol	28.40	153 (−)	123	✔	✔	✔	✔	✔	✔	✔		
*p*-Hydroxybenzoic acid	35.79	137 (−)	65, 93			✔	✔	✔	✔			
Chlorogenic acid	36.45	353 (−)	191				✔		✔			
Vanillic acid	39.59	167 (−)	123, 152	✔		✔						
Caffeic acid	40.13	179 (−)	79, 135	✔	✔	✔	✔	✔	✔	✔		
*m*-Hydroxybenzoic acid	40.33	137 (−)	93				✔		✔			
Syringic acid	41.26	197 (−)	123, 153			✔						
Vanillin	46.65	153 (+)	93	✔		✔				✔	✔	✔
*p*-Coumaric acid	46.95	163 (−)	93, 119	✔	✔	✔	✔	✔	✔	✔	✔	
Benzoic acid	47.16	123 (+)	77, 81	✔	✔	✔	✔	✔	✔	✔		
Syringaldehyde	48.22	183 (+)	95, 123	✔	✔	✔	✔	✔	✔	✔	✔	✔
Ferulic acid	48.88	195 (+)	145, 177	✔		✔				✔	✔	
*m*-Coumaric acid	49.93	163 (−)	93, 119							✔		
*o*-Coumaric acid	52.27	163 (−)	93, 119				✔					
3,4,5-Trimethoxycinnamic acid	58.33	239 (+)	193, 221			✔					✔	
**Phenylpropanoids**												
Salidroside	32.00	299 (−)	119, 89	✔	✔	✔	✔	✔	✔	✔	✔	✔
Verbascoside	46.40	623 (−)	161, 46	✔	✔	✔	✔	✔	✔	✔	✔	✔
**Secoiridoids**												
Nuezhenide	47.60	685 (−)	453, 523	✔	✔	✔	✔	✔	✔	✔	✔	✔
Oleuropein	51.70	539 (−)	275, 307	✔	✔	✔	✔	✔	✔	✔	✔	✔

Not detected: epicatechin, pelargonin, myricetin, spiraeoside, epigallocatechin gallate, quercitrin, epicatechin gallate, phlorizin, epigallocatechin, ellagic acid, epicatechin, genistein, citric acid, cinnamic acid, succinic acid, quinic acid, and tyrosol.

## Data Availability

The original contributions presented in this study are included in the article and/or Supplementary Material. Further inquiries can be directed to the corresponding authors.

## Supplementary Materials

The following supporting information can be downloaded at: https://www.mdpi.com/article/10.3390/ph19030447/s1, Table S1. Concentration and percentual composition of each fatty acid in olive stones after Soxhlet extraction. Data presented as mean ± SD values (*n* = 3); Table S2. Concentration and percentual composition of each fatty acid in olive stones after Folch extraction. Data presented as mean ± SD values (*n* = 3); Table S3. Details of the MRM conditions applied in the HPLC-DAD-MS/MS analysis; Figure S1. DAD chromatogram (A) and Total Ion Chromatogram in positive (B) and negative (C) ESI modes of MAE H2O extract; Figure S2. DAD chromatogram (A) and Total Ion Chromatogram in positive (B) and negative (C) modes of MAE EtOH extract; Figure S3. DAD chromatogram (A) and Total Ion Chromatogram in positive (B) and negative (C) ESI modes of MAE EtOH50 % extract; Figure S4. DAD chromatogram (A) and Total Ion Chromatogram in positive (B) and negative (C) ESI modes of MAE EtOH80% extract; Figure S5. DAD chromatogram (A) and Total Ion Chromatogram in positive (B) and negative (C) ESI modes of MAE MeOH extract; Figure S6. DAD chromatogram (A) and Total Ion Chromatogram in positive (B) and negative (C) ESI modes of MAE MeOH50% extract; Figure S7. DAD chromatogram (A) and Total Ion Chromatogram in positive (B) and negative (C) ESI modes of MAE MeOH50% extract.

## References

[1] Soares T.F., Alves R.C., Oliveira M.B.P.P. (2024). From Olive Oil Production to By-Products: Emergent Technologies to Extract Bioactive Compounds. Food Rev. Int..

[2] Xia M., Zhong Y., Peng Y., Qian C. (2022). Olive Oil Consumption and Risk of Cardiovascular Disease and All-Cause Mortality: A Meta-Analysis of Prospective Cohort Studies. Front. Nutr..

[3] Ilak Peršurić A.S., Težak Damijanić A. (2021). Connections between Healthy Behaviour, Perception of Olive Oil Health Benefits, and Olive Oil Consumption Motives. Sustainability.

[4] Martínez-González M.A., Sayón-Orea C., Bullón-Vela V., Bes-Rastrollo M., Rodríguez-Artalejo F., Yusta-Boyo M.J., García-Solano M. (2022). Effect of Olive Oil Consumption on Cardiovascular Disease, Cancer, Type 2 Diabetes, and All-Cause Mortality: A Systematic Review and Meta-Analysis. Clin. Nutr..

[5] Ghobadi S., Hassanzadeh-Rostami Z., Mohammadian F., Nikfetrat A., Ghasemifard N., Raeisi Dehkordi H., Faghih S. (2019). Comparison of Blood Lipid-Lowering Effects of Olive Oil and Other Plant Oils: A Systematic Review and Meta-Analysis of 27 Randomized Placebo-Controlled Clinical Trials. Crit. Rev. Food Sci. Nutr..

[6] International Olive Council World Market of Olive Oil and Table Olives 2024. https://www.internationaloliveoil.org/world-market-of-olive-oil-and-table-olives-data-from-december-2024/.

[7] Rivero-Pino F., Millan-Linares M.C., Villanueva-Lazo A., Fernandez-Prior Á., Montserrat-de-la-Paz S. (2024). In Vivo Evidences of the Health-Promoting Properties of Bioactive Compounds Obtained from Olive By-Products and Their Use as Food Ingredient. Crit. Rev. Food Sci. Nutr..

[8] El S.N., Karakaya S. (2009). Olive Tree (*Olea europaea*) Leaves: Potential Beneficial Effects on Human Health. Nutr. Rev..

[9] Silva S., Gomes L., Leitão F., Coelho A., Boas L. (2006). Phenolic Compounds and Antioxidant Activity of *Olea europaea* L. Fruits and Leaves. Food Sci. Technol. Int..

[10] Nicolì F., Negro C., Vergine M., Aprile A., Nutricati E., Sabella E., Miceli A., Luvisi A., De Bellis L. (2019). Evaluation of Phytochemical and Antioxidant Properties of 15 Italian *Olea europaea* L. Cultivar Leaves. Molecules.

[11] Zhang C., Xin X., Zhang J., Zhu S., Niu E., Zhou Z., Liu D. (2022). Comparative Evaluation of the Phytochemical Profiles and Antioxidant Potentials of Olive Leaves from 32 Cultivars Grown in China. Molecules.

[12] Ferreira D.M., Barreto-Peixoto J., Andrade N., Machado S., Silva C., Lobo J.C., Nunes M.A., Álvarez-Rivera G., Ibáñez E., Cifuentes A. (2024). Comprehensive Analysis of the Phytochemical Composition and Antitumoral Activity of an Olive Pomace Extract Obtained by Mechanical Pressing. Food Biosci..

[13] Abbattista R., Ventura G., Calvano C.D., Cataldi T.R., Losito I. (2021). Bioactive Compounds in Waste By-Products from Olive Oil Production: Applications and Structural Characterization by Mass Spectrometry Techniques. Foods.

[14] Rodríguez G., Lama A., Rodríguez R., Jiménez A., Guillén R., Fernández-Bolaños J. (2008). Olive Stone: An Attractive Source of Bioactive and Valuable Compounds. Bioresour. Technol..

[15] Contreras-Angulo L.A., Laaroussi H., Ousaaid D., Bakour M., Lyoussi B., Ferreira-Santos P. (2025). Sustainable Valorization of Olive Oil By-Products: Green Extraction of Phytochemicals, Encapsulation Strategies, and Food Applications. J. Food Sci..

[16] El Fessikh M., Elhrech H., El Idrissi A., Lee L., Al Abdulmonem W., El Omari N., Bouyahya A. (2025). Sustainable Valorization of Olive Stone By-Products: Opportunities and Challenges. J. Food Compos. Anal..

[17] Macarez A.C., Maalej H., Drobek M., Pochat-Bohatier C., Maalej A., Chamkha M., Li S. (2025). From Olive Stones Waste to Valuable Resource: Exploring Various Techniques for Cellulose Extraction. J. Environ. Chem. Eng..

[18] Ravindran R., Jaiswal A.K. (2016). Exploitation of Food Industry Waste for High-Value Products. Trends Biotechnol..

[19] Nunes M.A., Pimentel F.B., Costa A.S.G., Alves R.C., Oliveira M.B.P.P. (2016). Olive By-Products for Functional and Food Applications: Challenging Opportunities to Face Environmental Constraints. Innov. Food Sci. Emerg. Technol..

[20] Servili M., Baldioli M., Selvaggini R., Macchioni A., Montedoro G.F. (1999). Phenolic Compounds of Olive Fruit: One- and Two-Dimensional Nuclear Magnetic Resonance Characterization of Nüzhenide and Its Distribution in the Constitutive Parts of Fruit. J. Agric. Food Chem..

[21] Elbir M., Es-Safi N.E., Amhoud A., Mbarki M. (2015). Characterization of Phenolic Compounds in Olive Stones of Three Moroccan Varieties. Maderas Cienc. Tecnol..

[22] Maestri D., Barrionuevo D., Bodoira R., Zafra A., Jiménez-López J., de Dios Alché J. (2019). Nutritional Profile and Nutraceutical Components of Olive (*Olea europaea* L.) Seeds. J. Food Sci. Technol..

[23] Xie P., Cecchi L., Bellumori M., Balli D., Giovannelli L., Huang L., Mulinacci N. (2021). Phenolic Compounds and Triterpenes in Different Olive Tissues and Olive Oil By-Products, and Cytotoxicity on Human Colorectal Cancer Cells: The Case of Frantoio, Moraiolo and Leccino Cultivars (*Olea europaea* L.). Foods.

[24] Cecchi L., Ghizzani G., Bellumori M., Lammi C., Zanoni B., Mulinacci N. (2023). Virgin Olive Oil By-Product Valorization: An Insight into the Phenolic Composition of Olive Seed Extracts from Three Cultivars as Sources of Bioactive Molecules. Molecules.

[25] Gouvinhas I., Garcia J., Granato D., Barros A. (2022). Seed Phytochemical Profiling of Three Olive Cultivars, Antioxidant Capacity, Enzymatic Inhibition, and Effects on Human Neuroblastoma Cells (SH-SY5Y). Molecules.

[26] Obied H.K., Allen M.S., Bedgood D.R., Prenzler P.D., Robards K., Stockmann R. (2005). Bioactivity and Analysis of Biophenols Recovered from Olive Mill Waste. J. Agric. Food Chem..

[27] Alara O.R., Abdurahman N.H., Ukaegbu C.I. (2021). Extraction of Phenolic Compounds: A Review. Curr. Res. Food Sci..

[28] Kaufmann B., Christen P. (2002). Recent Extraction Techniques for Natural Products: Microwave-Assisted Extraction and Pressurised Solvent Extraction. Phytochem. Anal..

[29] Bitwell C., Sen I.S., Luke C., Kakoma M.K. (2023). A Review of Modern and Conventional Extraction Techniques and Their Applications for Extracting Phytochemicals from Plants. Sci. Afr..

[30] López-Salazar H., Camacho-Díaz B.H., Ocampo M.L.A., Jiménez-Aparicio A.R. (2023). Microwave-Assisted Extraction of Functional Compounds from Plants: A Review. BioResources.

[31] Garcia-Garcia G., Rahimifard S., Matharu A.S., Dugmore T.I.J. (2019). Life-Cycle Assessment of Microwave-Assisted Pectin Extraction at Pilot Scale. ACS Sustain. Chem. Eng..

[32] Catalano T., Selvaggi F., Cotellese R., Aceto G.M. (2025). The Role of Reactive Oxygen Species in Colorectal Cancer Initiation and Progression: Perspectives on Theranostic Approaches. Cancers.

[33] Ranalli A., Pollastri L., Contento S., Di Loreto G., Iannucci E., Lucera L., Russi F. (2002). Acylglycerol and Fatty Acid Components of Pulp, Seed, and Whole Olive Fruit Oils: Their Use to Characterize Fruit Variety by Chemometrics. J. Agric. Food Chem..

[34] Hannachi H., Benabderrahim M.A., Elfalleh W., Wang R., Ying M. (2020). Amino and Fatty Acids Composition of Olive Stones for the Discrimination of *Olea europaea* subsp. *europaea* Varieties. Mediterr. Bot..

[35] Revelou P.-K., Xagoraris M., Alexandropoulou A., Kanakis C.D., Papadopoulos G.K., Pappas C.S., Tarantilis P.A. (2021). Chemometric Study of Fatty Acid Composition of Virgin Olive Oil from Four Widespread Greek Cultivars. Molecules.

[36] Jimenez-Lopez C., Carpena M., Lourenço-Lopes C., Gallardo-Gomez M., Lorenzo J.M., Barba F.J., Prieto M.A., Simal-Gandara J. (2020). Bioactive Compounds and Quality of Extra Virgin Olive Oil. Foods.

[37] Li Y., Hruby A., Bernstein A.M., Ley S.H., Wang D.D., Chiuve S.E., Sampson L., Rexrode K.M., Rimm E.B., Willett W.C. (2015). Saturated Fats Compared with Unsaturated Fats and Sources of Carbohydrates in Relation to Risk of Coronary Heart Disease. J. Am. Coll. Cardiol..

[38] Osei J.B.D., Amiri A., Wang J., Tavares M.T., Kiatkittipong W., Najdanovic-Visak V. (2022). Recovery of Oils and Antioxidants from Olive Stones. Biomass Bioenergy.

[39] Alu’datt M., Alli I., Ereifej K., Alhamad M., Alsaad A., Rababah T. (2011). Optimisation and Characterisation of Various Extraction Conditions of Phenolic Compounds and Antioxidant Activity in Olive Seeds. Nat. Prod. Res..

[40] Bastola K.P., Guragain Y.N., Bhadriraju V., Vadlani P.V. (2017). Evaluation of Standards and Interfering Compounds in the Determination of Phenolics by Folin–Ciocalteu Assay Method for Effective Bioprocessing of Biomass. Am. J. Anal. Chem..

[41] Shi L., Zhao W., Yang Z., Subbiah V., Suleria H.A.R. (2022). Extraction and Characterization of Phenolic Compounds and Their Potential Antioxidant Activities. Environ. Sci. Pollut. Res..

[42] Schmidt L., Prestes O.D., Augusti P.R., Fonseca Moreira J.C. (2023). Phenolic Compounds and Contaminants in Olive Oil and Pomace—A Narrative Review of Their Biological and Toxic Effects. Food Biosci..

[43] Abaza L., Taamalli A., Nsir H., Zarrouk M. (2015). Olive Tree (*Olea europaea* L.) Leaves: Importance and Advances in the Analysis of Phenolic Compounds. Antioxidants.

[44] Khwaldia K., Attour N., Matthes J., Beck L., Schmid M. (2022). Olive Byproducts and Their Bioactive Compounds as a Valuable Source for Food Packaging Applications. Compr. Rev. Food Sci. Food Saf..

[45] Romani A., Ieri F., Urciuoli S., Noce A., Marrone G., Nediani C., Bernini R. (2019). Health Effects of Phenolic Compounds Found in Extra-Virgin Olive Oil, By-Products, and Leaf of *Olea europaea* L. Nutrients.

[46] Sasaki M., Manalu H.T., Kamogawa R., Issasi C.S.C., Quitain A.T., Kida T. (2023). Fast and Selective Production of Quercetin and Saccharides from Rutin Using Microwave-Assisted Hydrothermal Treatment in the Presence of Graphene Oxide. Food Chem..

[47] Safarzadeh Markhali F. (2021). Effect of Processing on Phenolic Composition of Olive Oil Products and Olive Mill By-Products and Possibilities for Enhancement of Sustainable Processes. Processes.

[48] Maestro-Durán R., Cabello R., Ruiz-Gutierrez V., Fiestas P., Vázquez-Roncero A. (1994). Glucósidos fenólicos amargos de las semillas del olivo (*Olea europaea*). Grasas Aceites.

[49] Ryan D., Antolovich M., Herlt T., Prenzler P.D., Lavee S., Robards K. (2002). Identification of Phenolic Compounds in Tissues of the Novel Olive Cultivar Hardy’s Mammoth. J. Agric. Food Chem..

[50] Silva S., Gomes L., Leitão F., Bronze M., Coelho A., Boas L. (2010). Secoiridoids in Olive Seed: Characterization of Nüzhenide and 11-Methyl Oleosides by Liquid Chromatography with Diode Array and Mass Spectrometry. Grasas Aceites.

[51] Mansour A., Porter E., Kite G., Simmonds M., Abdelhédi R., Bouaziz M. (2015). Phenolic Profile Characterization of Chemlali Olive Stones by Liquid Chromatography–Ion Trap Mass Spectrometry. J. Agric. Food Chem..

[52] González-Hidalgo I., Bañón S., Ros J.M. (2012). Evaluation of Table Olive By-Product as a Source of Natural Antioxidants. Int. J. Food Sci. Technol..

[53] Flores G., Blanch G.P., Ruiz del Castillo M.L. (2018). Abscisic Acid Treated Olive Seeds as a Natural Source of Bioactive Compounds. LWT.

[54] Bai L., Xu D., Zhou Y.-M., Zhang Y.-B., Zhang H., Chen Y.-B., Cui Y.-L. (2022). Antioxidant Activities of Natural Polysaccharides and Their Derivatives for Biomedical and Medicinal Applications. Antioxidants.

[55] Diogo J. (2013). Valorization of Wild Olives (Olea europaea var. sylvestris) as Potential Source of Functional Ingredients.

[56] Moudache M., Colon M., Nerín C., Zaidi F. (2016). Phenolic Content and Antioxidant Activity of Olive By-Products and Antioxidant Film Containing Olive Leaf Extract. Food Chem..

[57] Katsinas N., Bento da Silva A., Enríquez-de-Salamanca A., Fernández N., Bronze M.R., Rodríguez-Rojo S. (2021). Pressurized Liquid Extraction Optimization from Supercritical Defatted Olive Pomace: A Green and Selective Phenolic Extraction Process. ACS Sustain. Chem. Eng..

[58] (2009). Biological Evaluation of Medical Devices—Part 5: Tests for In Vitro Cytotoxicity.

[59] Neira-Ospina E., Rojas J., Santa-Gonzalez G.A., Gil Garzón M.A. (2024). Phenolic compounds in grapes (genus Vitis): A review of their antioxidant activity, antiproliferative capacity, and cytotoxic effect on colorectal cancer. J. Appl. Pharm. Sci..

[60] Fink H., Langselius O., Vignat J., Rumgay H., Rehm J., Martinez R.X., Santero M., Lopez-Perez L., Inoue M., Zeng H. (2026). Global and Regional Cancer Burden Attributable to Modifiable Risk Factors to Inform Prevention. Nat. Med..

[61] López-Gómez L., Uranga J.A. (2024). Polyphenols in the Prevention and Treatment of Colorectal Cancer: A Systematic Review of Clinical Evidence. Nutrients.

[62] Tan B.L., Zulkifli F., Norhaizan M.E. (2025). Dietary Polyphenols as Modulators of Cell Signaling and Inflammation in Colorectal Carcinogenesis. Front. Nutr..

[63] Cárdeno A., Sánchez-Hidalgo M., Rosillo M.A., de la Lastra C.A. (2013). Oleuropein, a Secoiridoid Derived from Olive Tree, Inhibits the Proliferation of Human Colorectal Cancer Cells through Downregulation of HIF-1α. Nutr. Cancer.

[64] Rishmawi S., Haddad F., Dokmak G., Karaman R. (2022). A Comprehensive Review on the Anti-Cancer Effects of Oleuropein. Life.

[65] Almatroodi S.A., Almatroudi A., Alharbi H.O.A., Khan A.A., Rahmani A.H. (2024). Effects and Mechanisms of Luteolin, a Plant-Based Flavonoid, in the Prevention of Cancers via Modulation of Inflammation and Cell Signaling Molecules. Molecules.

[66] Naponelli V., Piscazzi A., Mangieri D. (2025). Cellular and Molecular Mechanisms Modulated by Genistein in Cancer. Int. J. Mol. Sci..

[67] Zuo Q., Wu R., Xiao X., Yang C., Yang Y., Wang C., Lin L., Kong A.-N. (2018). The Dietary Flavone Luteolin Epigenetically Activates the Nrf2 Pathway and Blocks Cell Transformation in Human Colorectal Cancer HCT116 Cells. J. Cell. Biochem..

[68] Shafiee G., Saidijam M., Tavilani H., Ghasemkhani N., Khodadadi I. (2016). Genistein Induces Apoptosis and Inhibits Proliferation of HT29 Colon Cancer Cells. Int. J. Mol. Cell Med..

[69] Moussaoui R., Labbaci W., Hemar N., Ahcèneyouyou A., Youcef A. (2008). Physico-Chemical Characteristics of Oils Extracted from Three Compartments of the Olive Fruit (Pulp, Endocarp and Seed) of Variety Chemlal Cultivated in Kabylia (Algeria). J. Food Agric. Environ..

[70] Alves E., Rey F., Melo T., Barros M.P., Domingues P., Domingues R. (2022). Bioprospecting Bioactive Polar Lipids from Olive (*Olea europaea* cv. *Galega vulgar*) Fruit Seeds: LC–HR–MS/MS Fingerprinting and Sub-Geographic Comparison. Foods.

[71] Folch J., Lees M., Stanley G.H.S. (1957). A Simple Method for the Isolation and Purification of Total Lipides from Animal Tissues. J. Biol. Chem..

[72] Serra A.T., Silva S.D., Pleno de Gouveia L., Alexandre A.M.R.C., Pereira C.V., Pereira A.B., Partidário A.C., Silva N.E., Bohn T., Gonçalves V.S.S. (2021). A Single Dose of Marine *Chlorella vulgaris* Increases Plasma Concentrations of Lutein, β-Carotene and Zeaxanthin in Healthy Male Volunteers. Antioxidants.

[73] Quaresma M.A.G., Antunes I.C., Ferreira B.G., Parada A., Elias A., Barros M., Santos C., Partidário A., Mourato M., Roseiro L.C. (2022). The Composition of the Lipid, Protein and Mineral Fractions of Quail Breast Meat Obtained from Wild and Farmed Specimens of Common Quail (*Coturnix coturnix*) and Farmed Japanese Quail (*Coturnix japonica domestica*). Poult. Sci..

[74] Weng V., Cardeira M., Bento-Silva A., Serra A.T., Brazinha C., Bronze M.R. (2023). Arabinoxylan from Corn Fiber Obtained through Alkaline Extraction and Membrane Purification: Relating Bioactivities with the Phenolic Compounds. Molecules.

[75] Huang D., Ou B., Hampsch-Woodill M., Flanagan J.A., Prior R.L. (2002). High-Throughput Assay of Oxygen Radical Absorbance Capacity (ORAC) Using a Multichannel Liquid Handling System Coupled with a Microplate Fluorescence Reader in 96-Well Format. J. Agric. Food Chem..

[76] Moore J., Yin J.-J., Yu L. (2006). Novel Fluorometric Assay for Hydroxyl Radical Scavenging Capacity (HOSC) Estimation. J. Agric. Food Chem..

[77] Oliveira-Alves S.C., Andrade F., Prazeres I., Silva A.B., Capelo J., Duarte B., Caçador I., Coelho J., Serra A.T., Bronze M.R. (2021). Impact of Drying Processes on the Nutritional Composition, Volatile Profile, Phytochemical Content and Bioactivity of *Salicornia ramosissima* J. Woods. Antioxidants.

[78] Gagnon M., Zihler Berner A., Chervet N., Chassard C., Lacroix C. (2013). Comparison of the Caco-2, HT-29 and the Mucus-Secreting HT29-MTX Intestinal Cell Models to Investigate *Salmonella* Adhesion and Invasion. J. Microbiol. Methods.

[79] Nollevaux G., Devillé C., El Moualij B., Zorzi W., Deloyer P., Schneider Y.-J., Peulen O., Dandrifosse G. (2006). Development of a Serum-Free Co-Culture of Human Intestinal Epithelium Cell Lines (Caco-2/HT29-5M21). BMC Cell Biol..

[80] Sullivan L.M., Weinberg J., Keaney J.F. (2016). Common Statistical Pitfalls in Basic Science Research. J. Am. Heart Assoc..

[81] Saini K., Prasad P., Shang X., Keum Y.-S. (2021). Advances in Lipid Extraction Methods—A Review. Int. J. Mol. Sci..

