# Supplementary material for: Valorization of Olive Stones: Chemical Composition and Bioactivity
Source: Pharmaceuticals (Basel). 2026 Mar 10;19(3):447. doi: 10.3390/ph19030447 (PMC13028983; doi:10.3390/ph19030447)
Supplement: Supplementary file 1 [file pharmaceuticals-19-00447-s001.zip › pharmaceuticals-4162865-supplementary.pdf]

Valorization of Olive Stones: Chemical Composition and Bioactivity

Bruna Nunes[1], Naiara Fernandéz[2], Andreia Bento Silva[1], Ana Partidário[3], Joana Marto[1], Elizabete Alexandre[4], Sofia Lourenço[5], Teresa Serra[1,6], Maria Rosário Bronze[1,2,6], Noélia Duarte[1,*], Ana Margarida Rodrigues[2,*]

Supporting Information

Supplementary Table S1 – Concentration and percentual composition of each fatty acid in olive stones after Soxhlet extraction. Data presented as mean ± SD values (n = 3)

Fatty acid	*Average concentration (µg/mg of extract)*	*Average % composition*
Myristic acid (C14)	0.49 ± 0.09	0.09 ± 0.01
Palmitic acid (C16)	89.22 ± 4.55	16.63 ± 0.29
Trans-palmitoleic acid (C16:1 *trans*)	0.33 ± 0.06	0.06 ± 0.01
Palmitoleic acid (C16:1 *cis*-7)	0.97 ± 0.05	0.18 ± 0.00
Palmitoleic acid (C16:1 *cis*-9)	5.27 ± 0.27	0.98 ± 0.02
Margaric acid (C17)	0.79 ± 0.11	0.15 ± 0.01
Heptadecenoic acid (C17:1)	0.81 ± 0.11	0.15 ± 0.01
Stearic acid (C18)	12.59 ± 1.00	2.34 ± 0.05
Oleic acid (C18:1 *cis*-9)	349.30 ± 24.54	65.03 ± 0.24
Linoleic acid (C18:2)	59.86 ± 3.95	11.15 ± 0.02
Linolenic acid (C18:3)	3.40 ± 0.25	0.63 ± 0.01
Arachidic acid (C20)	4.56 ± 0.40	0.85 ± 0.02
Eicosenoic acid (C20:1 *cis*-5)	1.64 ± 0.00	0.32 ± 0.00
Eicosenoic acid (C20:1 *cis*-9)	1.60 ± 0.16	0.30 ± 0.03
Gadoleic acid (C20:1 *cis*-11)	3.11 ± 0.30	0.58 ± 0.05
Eicosadienoic acid (C20:2)	1.25 ± 0.11	0.23 ± 0.01
Behenic acid (C22)	3.09 ± 0.27	0.58 ± 0.03
Docosadienoic acid (C22:2)	0.87 ± 0.00	0.15 ± 0.00

Supplementary Table S2 – Concentration and percentual composition of each fatty acid in olive stones after Folch extraction. Data presented as mean ± SD values (*n* = 3)

Fatty acid	*Average concentration (µg/mg of extract)*	*Average % composition*
Myristic acid (C14)	0.34 ± 0.05	0.10 ± 0.01
Palmitic acid (C16)	62.71 ± 2.18	19.20 ± 0.37
Trans-palmitoleic acid (C16:1 *trans*)	0.66 ± 0.00	0.20 ± 0.00
Palmitoleic acid (C16:1 *cis*-9)	2.96 ± 0.09	0.91 ± 0.02
Margaric acid (C17)	0.61 ± 0.04	0.19 ± 0.00
Heptadecenoic acid (C17:1)	0.53 ± 0.06	0.16 ± 0.02
Stearic acid (C18)	9.03 ± 0.34	2.76 ± 0.05
Oleic acid (C18:1 *cis*-9)	194.73 ± 8.58	59.61 ± 0.26
Vaccenic acid (C18:1 *cis*-11)	6.38 ± 0.25	1.99 ± 0.01
Linoleic acid (C18:2)	28.12 ± 0.87	8.61 ± 0.16
Linolenic acid (C18:3)	1.42 ± 0.12	0.43 ± 0.03
Arachidic acid (C20)	3.21 ± 0.32	0.98 ± 0.09
Eicosenoic acid (C20:1 *cis*-9)	4.45 ± 0.25	1.39 ± 0.01
Gadoleic acid (C20:1 *cis*-11)	8.94 ± 0.68	2.73 ± 0.10
Behenic acid (C22)	1.83 ± 0.12	0.56 ± 0.01

Supplementary Table S3. Details of the MRM conditions applied in the HPLC-DAD-MS/MS analysis.

Compounds	*m/z*	MS/MS ions	Cone Voltage (V)	Collision Energy (eV)
Cyanidin 3-o-rutinoside (+)	595	287, 449	40	20
Luteolin (-)	285	107, 133	50	30
Apigenin (+)	271	119, 153	50	30
3,4,5-trimethoxycinnamic acid (+)	239	193, 221	20	10
Vanillin (+)	153	93	20	10
Hesperidin (-)	609	228, 301	50	20
Gallocatechin gallate (-)	457	125, 169	30	15
Luteolin 7-glucoside	447	284, 285	50	20
Catechin gallate (-)	441	169, 289	40	20
Chlorogenic acid (-)	353	191	40	15
Isorhamnetin (-)	315	151, 300	40	20
Quercetin (-)	301	151, 179	40	20
Catechin (+)	291	123, 139	20	10
Phloretin (-)	273	123, 167	40	15
Ferulic acid (+)	195	145, 177	10	10
Caffeic acid (-)	179	79, 135	30	12
Vanillic acid (-)	167	123, 152	20	12
o-coumaric acid (-)	163	93, 119	20	10
Cinnamic acid (+)	149	103, 131	10	10
m-hydroxybenzoic acid (-)	137	93	20	10
Succinic acid (-)	117	73, 99	20	10
Epicatechin (+)	291	123, 139	20	10
Pelargonin (+)	595	271, 433	40	20
Myricetin (+)	319	153, 217	50	30
Kaempferol (+)	287	121, 153	50	30

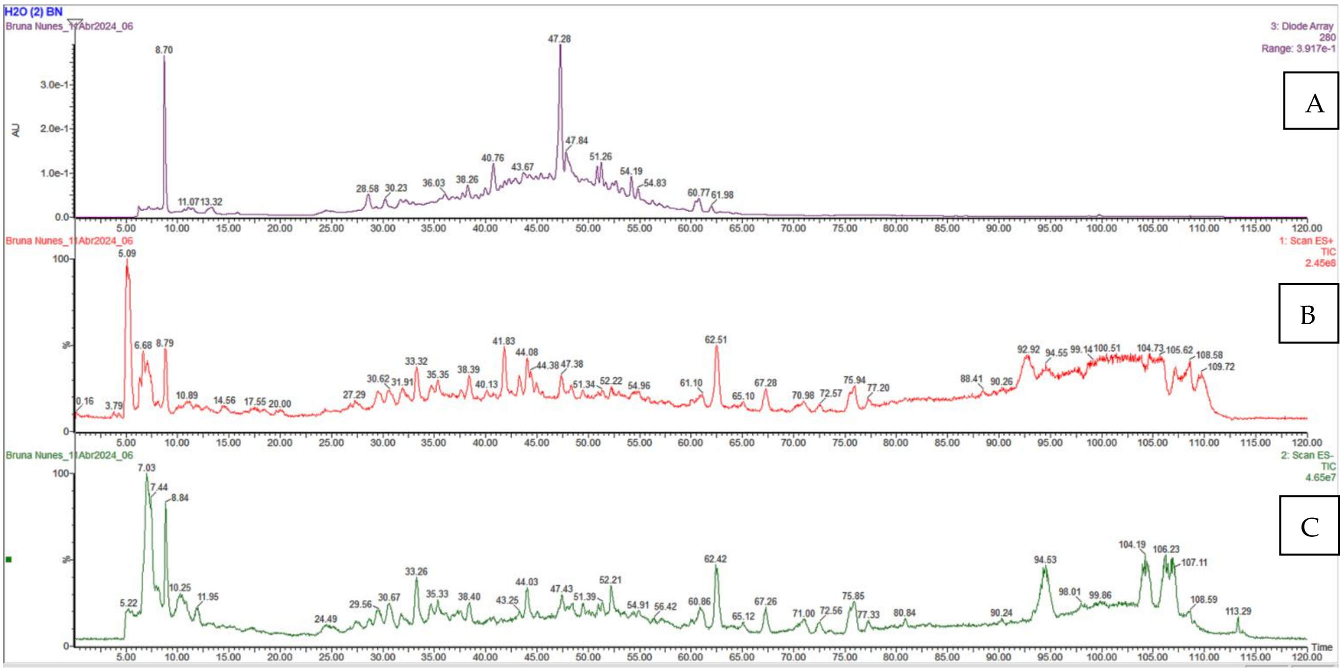

Supplementary Figure S1. DAD chromatogram (A) and Total Ion Chromatogram in positive (B) and negative (C) ESI modes of MAE H_2O extract.

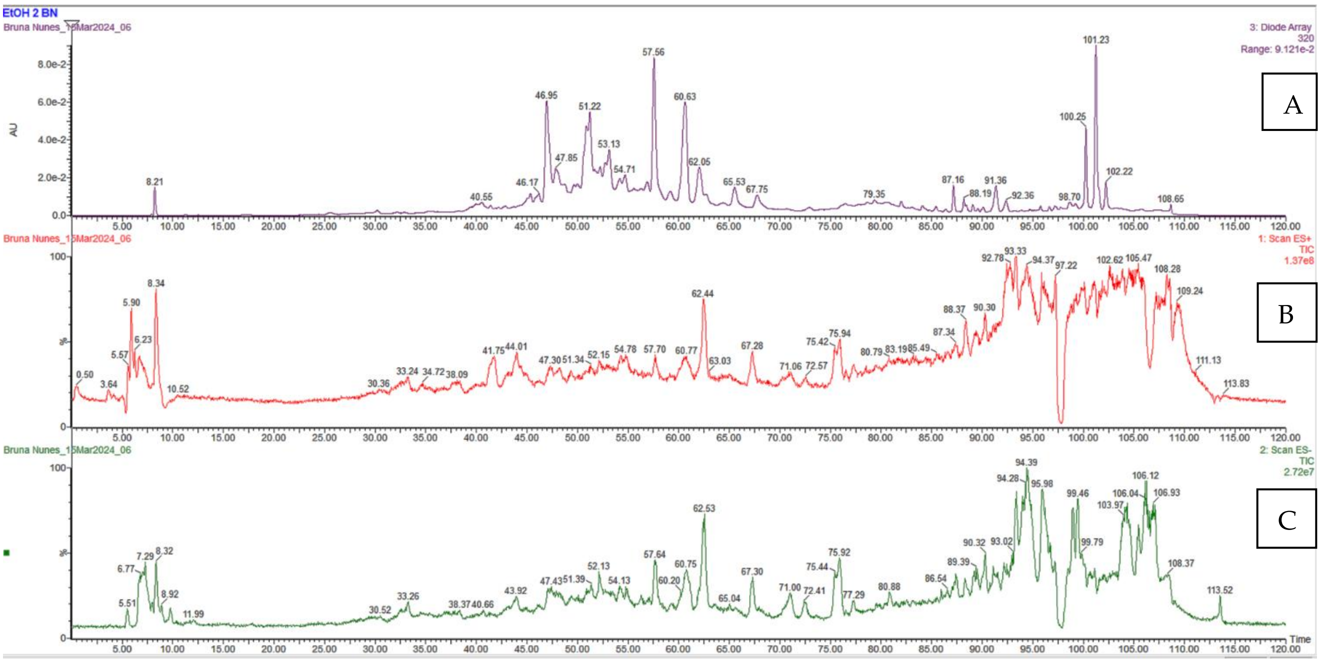

Supplementary Figure S2. DAD chromatogram and Total Ion Chromatogram in positive (B) and negative (C) modes of MAE EtOH extract.

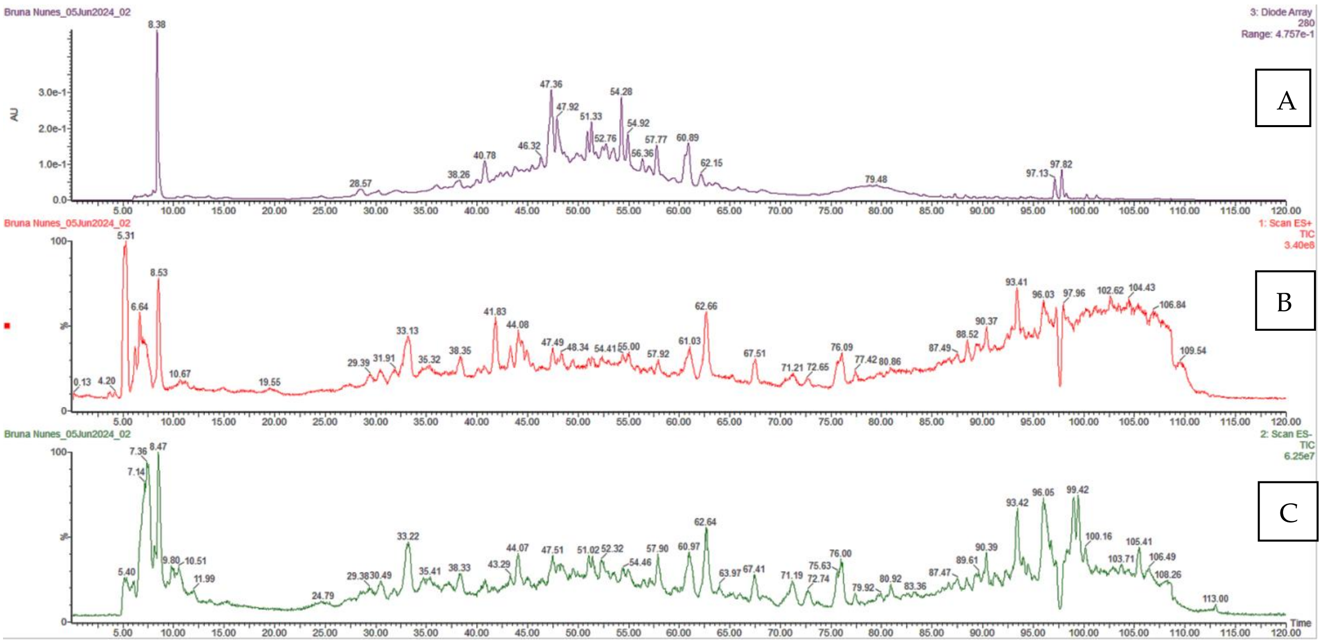

Supplementary Figure S3. DAD chromatogram (A) and Total Ion Chromatogram in positive (B) and negative (C) ESI modes of MAE EtOH50 % extract.

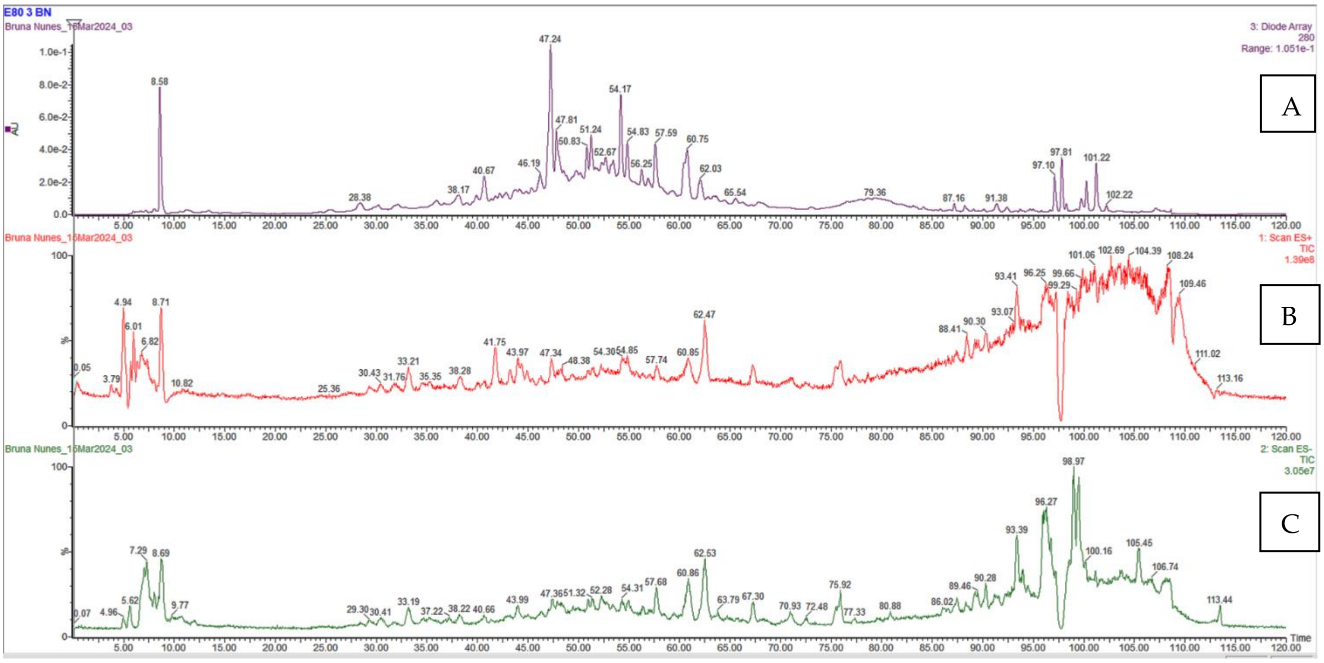

Supplementary Figure S4. DAD chromatogram (A) and Total Ion Chromatogram in positive (B) and negative (C) ESI modes of MAE EtOH80% extract.

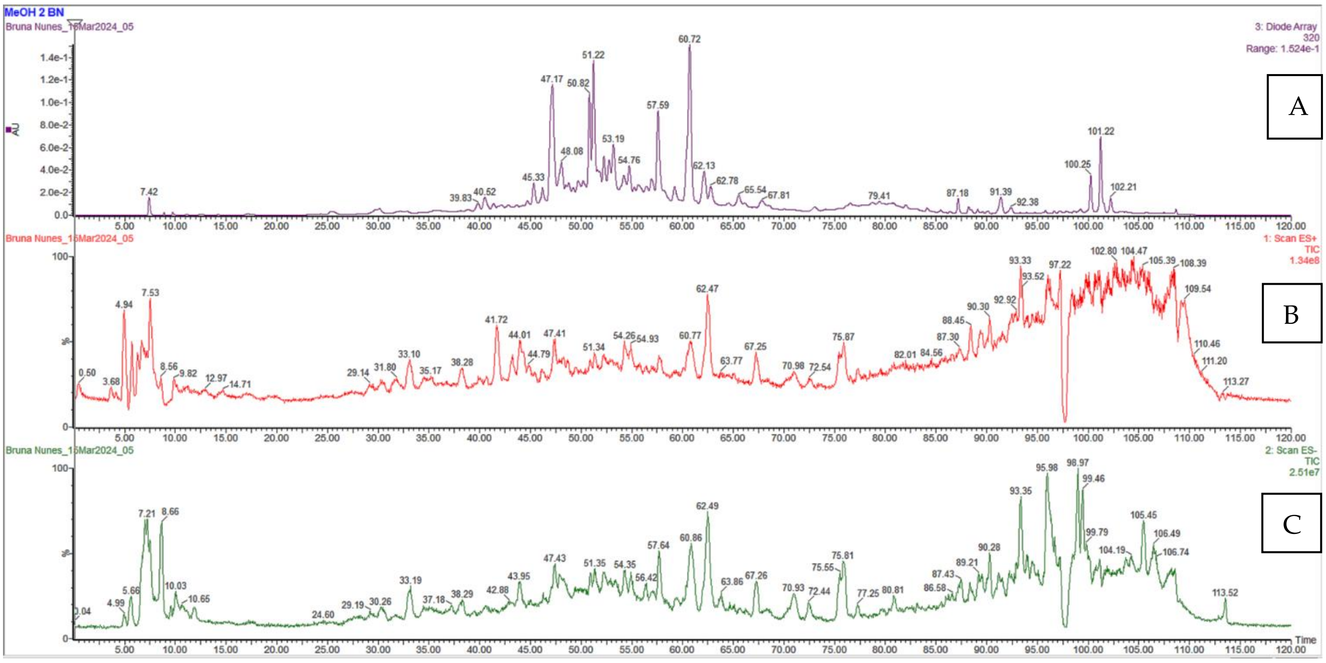

Supplementary Figure S5. DAD chromatogram (A) and Total Ion Chromatogram in positive (B) and negative (C) ESI modes of MAE MeOH extract.

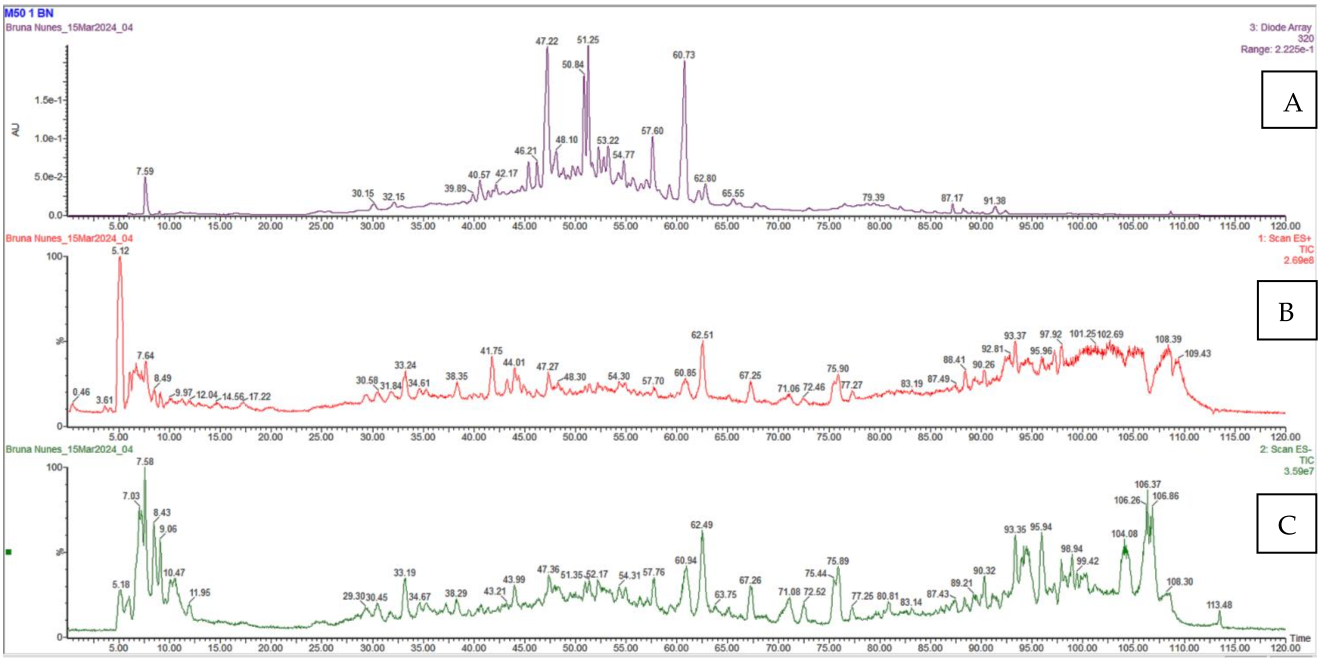

Supplementary Figure S6. DAD chromatogram (A) and Total Ion Chromatogram in positive (B) and negative (C) ESI modes of MAE MeOH50% extract.

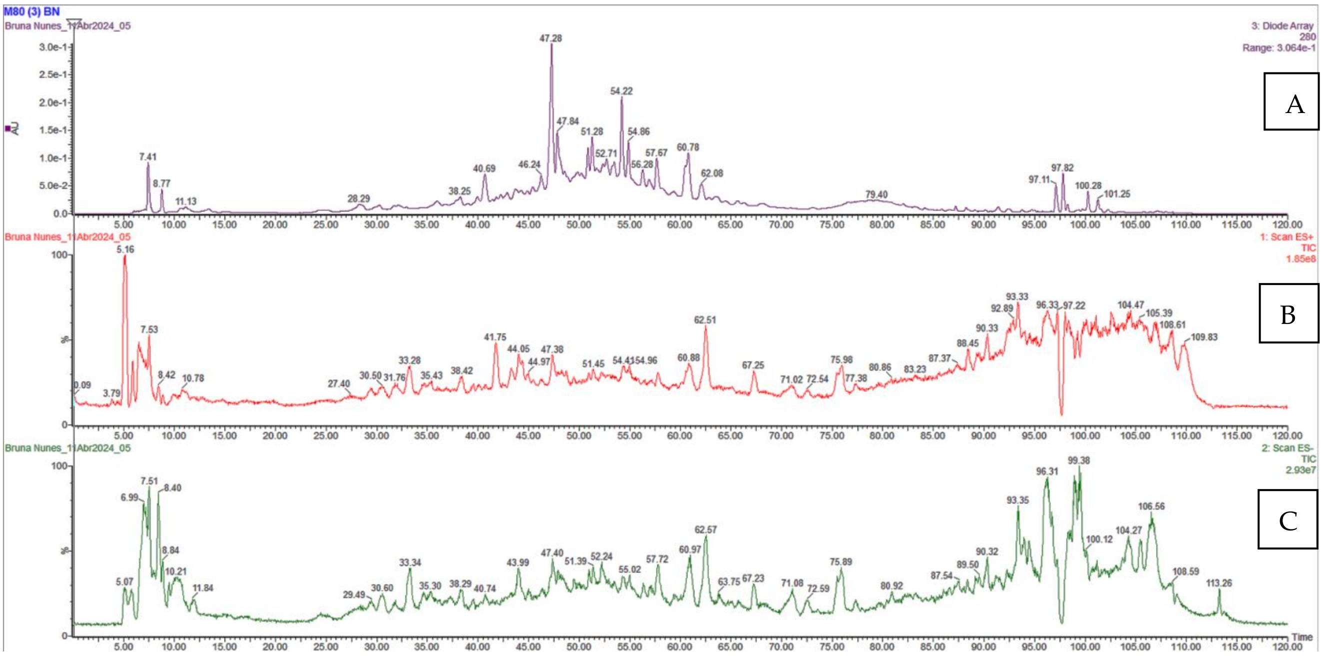

Supplementary Figure S7. DAD chromatogram (A) and Total Ion Chromatogram in positive (B) and negative (C) ESI modes of MAE MeOH50% extract.
